# Nutrient patterns in relation to metabolic health status in overweight and obese adolescents

**DOI:** 10.1038/s41598-023-27510-w

**Published:** 2023-01-04

**Authors:** Parisa Rouhani, Saeideh Mirzaei, Ali Asadi, Masoumeh Akhlaghi, Parvane Saneei

**Affiliations:** 1 Department of Community Nutrition, School of Nutrition and Food Science, Nutrition and Food Security Research Center, Students’ Research Committee, Isfahan University of Medical Sciences, Isfahan, Iran; 2 Department of Community Nutrition, School of Nutrition and Food Science, Shiraz University of Medical Sciences, Shiraz, Iran; 3 Department of Exercise Physiology, School of Physical Education and Sport Sciences, University of Tehran, Tehran, Iran; 4 Department of Community Nutrition, School of Nutrition and Food Science, Nutrition and Food Security Research Center, Isfahan University of Medical Sciences, PO Box 81745-151, Isfahan, Iran

**Keywords:** Nutrition, Paediatrics, Public health, Weight management

## Abstract

The association between dietary nutrient patterns (NPs) and metabolic health status has not been investigated in adolescents. This study aimed to evaluate the relationship between NPs and metabolic health status in Iranian adolescents with overweight and obesity. In this cross-sectional study, 203 obese/overweight adolescents were selected using a multistage mass random sampling method. To assess usual dietary intakes, a validated food frequency questionnaire was applied. Data of anthropometric and blood pressure were collected. Insulin, lipid profile, and glucose levels were determined using fasting blood samples. Two approaches [International Diabetes Federation (IDF) and a combination of IDF with Homeostasis Model Assessment Insulin Resistance (HOMA-IR)] were applied to identify metabolically healthy obese and metabolically unhealthy obese (MUO) adolescents. Participants had a mean age of 13.9 ± 1.61 years and 52.2% of them were girls. Three NPs were identified and labeled as "high minerals and vitamins" (NP1), "high carbohydrate" (NP2) and "high fat and sodium" (NP3). After adjustments for all potential confounders, no significant association was observed between higher adherence to NP1 and NP2 and odds of MUO; however, greater adherence to "high fat and sodium" NP was associated with higher odds of being MUO based on IDF (OR = 3.12; 95% CI 1.19, 8.09) and IDF/HOMA-IR (OR = 2.81; 95% CI 1.02, 7.74) definitions. Stratified analysis revealed that these associations were stronger in boys (versus girls) and obese (versus overweight) adolescents. In conclusion, high adherence to a "high fat and sodium" nutrient pattern was related to elevated chance of being MUO in Iranian adolescents, especially in boys and obese individuals. Therefore, less consumption of trans fatty acids, saturated fatty acids and sodium could be recommended to prevent MUO prevalence especially in boys with obesity.

## Introduction

Obesity is a chronic metabolic disorder caused by up normal fat aggregation in the body. Over the last few decades, the prevalence of obesity in children has increased globally^1,2^, with a noticeable increase in developing nations^3^. The global burden of disease (GBD) has predicted that approximately 124 million and 268 million of pediatrics will be respectively obese or overweight by the year of 2025^4^. Previous researches have estimated that about 4 million Iranian children and adolescents will expose overweight by the year of 2025^5^. Childhood overweight or obesity is a global public health challenge associated with serious childhood complications such as fatty liver diseases, insulin resistance, hypertension, and dyslipidemia which all can result in an increased risk of future cardiovascular disease (CVD), type 2 diabetes mellitus, and even mortality worldwide, and imposes a significant financial burden on healthcare systems^6,7^.

Previous evidence indicated that obesity and overweight in childhood could also enhance the risk of childhood diseases such as asthma, hypertension, abnormal glucose intolerance, sleep apnea, and diabetes mellitus^8,9^. These mentioned complications have not been developed in all adolescents with excess body weight; 18–44% of individuals with obesity might be free from cardio-metabolic risk factors (CMRFs)^10,11^. Overweight or obese individuals without CMRFs such as hypertension, dyslipidemia, insulin resistance, or prediabetes are referred to as metabolically healthy overweight or obese (MHO) individuals^12^ and those with the mentioned risk factors are referred to as metabolically unhealthy overweight or obese (MUO)^13,14^.

Dietary intake is one of the major determinants of metabolic health status. High intake of fresh fruits and vegetables^15^, vitamins^16^, antioxidants^17^, poultry, and fish^18,19^ has been associated with lower odds of MUO in pediatrics. On the other hand, poor diet quality and sedentary lifestyle have been announced as crucial risk factors for metabolic-syndrome (MetS) and obesity in children^20^. Previous evidence revealed that the nutritional intervention with the Mediterranean diet had beneficial effects on CMRFs in children with obesity^20–22^. Various dietary patterns include different kinds of nutrients in different populations. Thus, nutrient patterns can provide an easier way to compare dietary intakes between societies, because no matter what foods or food groups are consumed, the component nutrients in foods or food groups are the same^23^.

Most prior investigations on dietary intake in relation to metabolic complications in adolescents have focused on foods, food groups, or dietary patterns^12,21,22,24^, and less attention has been given to nutrient patterns. Furthermore, findings of these previous investigations on the relationship between dietary intakes and health status in adolescents were conflicting^21,22,24^. To the best of our knowledge, no previous study has investigated the relationship between patterns of nutrient intake and metabolic health status in adolescents with obesity. Therefore, this cross-sectional study aimed to investigate the linkage between patterns of nutrients intake and metabolic health status among Iranian overweight or obese adolescents.

## Methods

### Study design and population

This cross-sectional study was carried out on 203 school adolescents (102 girls and 101 boys) from 5 different educational districts of Isfahan, Iran, in 2020. To obtain enough MUO cases and investigate the relation of MUO and NPs, only adolescents with overweight and obesity were selected to be included in the current study. The estimated sample size based on a 60% prevalence of MUO among Iranian overweight and obese adolescents^25^, power of 80%, desired confidence interval of 0.95, type I error of 0.05, and precision (d) of 7% was 188. Participants between the ages of 12 and 18 years were chosen using a multistage mass sampling approach. Sixteen schools were randomly selected and body weight (kg) and height (cm) of all students of these schools were measured. Body mass index (BMI) was calculated according to the Quetelet formula (kg/m^2^); then, adolescents were classified as normal-weight, overweight, and obese^13,26^. In this way, obese and overweight individuals with various socioeconomic statuses were included in the study. Subjects with endocrine or genetic disorders (hypothyroidism, type 1 diabetes mellitus, and Cushing's syndrome) were not included in this investigation. Additionally, individuals on a weight-loss diet, or those who were taking supplements of minerals and vitamins, or drugs that might affect their blood glucose, lipid profile, body weight, or blood pressure were not eligible for this study. All participants and their parents signed an informed consent. The study protocol was ethically approved by Isfahan University of Medical Sciences (no. 2400212).

### Assessment of dietary intakes

Dietary intakes of participants in the preceding year was measured through a validated 147-item food frequency questionnaire (FFQ)^27^. Prior researches have proven that this FFQ could accurately indicate long-term dietary intakes and their relations with various diseases among Iranian adolescents^28,29^. Thus, this instrument could have a reasonable validity and reliability for assessing foods and nutrients in Iranian adolescents. A trained dietitian has completed the questionnaires and asked the individuals to report their food consumption frequency (on a basis of daily, weekly, or monthly) and amount consumed (on a basis of common standard portion sizes) in the past year. Then, using household measurements, all reported values were converted to gram per day^30^. Total energy and nutrient intakes of each individual were then calculated by summing up energy and nutrients of all food items. To derive nutrient intakes, the grams of food consumption were entered into the Nutritionist IV software. This software was based on the USDA food composition database.

### Assessment of anthropometric indices and cardiometabolic risk factors

A trained nutritionist has measured anthropometric indices of all participants. Weight was measured with a calibrated electronic scale (Seca Instruments, Germany) (nearest 0.1 kg). Standing height was measured with a stadiometer (nearest 0.1 cm). BMI was calculated as weight/(height)^2^ (kg/m^2^). Then, subjects were classified as normal, overweight, or obese adolescents, according to the age- and sex- specific World Health Organization (WHO) percentile cut-off points for adolescents^26^. Waist circumference (WC) was twice measured for each participant and the mean value of two assessments was considered as WC. After a resting period of 5 min, diastolic blood pressure (DBP) and systolic blood pressure (SBP) were twice measured with a 15 min recovery interval, at the right arm. The average of two measurements was considered in the analysis. To determine biochemical values, venous blood samples were obtained in a sitting position after a twelve-hour fasting, according to the standard protocol. The blood samples were collected in vacuum tubes and centrifuged within 30–45 min after collection. Fasting blood glucose (FBG) concentration was measured with an enzymatic colorimetric method by the use of glucose oxidase (Pars Azmoon commercial kits, Tehran, Iran). High-density lipoprotein cholesterol (HDL-c) was assayed by phosphotungstic acid, after precipitation of the apolipoprotein B-containing lipoproteins (Pars Azmoon commercial kits, Tehran, Iran). Serum triglyceride concentrations (TG) were also assayed using triacylglycerol kits by enzymatic colorimetric tests with glycerol phosphate oxidase (Pars Azmoon commercial kits, Tehran, Iran). Insulin concentrations were measured using ELISA kits (Diagnostic Biochem Canada Inc.). To estimate insulin resistance, we calculated Homeostasis Model Assessment Insulin Resistance (HOMA-IR) using the following formula: HOMA-IR = [insulin (µUI/mL) × glucose (mg/dL)]/405.

### Assessment of metabolic status

We applied two methods to classify participants into MUO and MHO. The first method was on the basis of the modified International Diabetes Federation (IDF) criteria^31^, by which students with two or more of the following CMRFs were considered as MUO: (1) increased triglycerides (≥ 150 mg/dL), (2) elevated fasting blood glucose (≥ 100 mg/dL), (3) decreased HDL-c (< 40 mg/dL for the age of < 16 y, and < 50 mg/dL in girls/ < 40 mg/dL in boys for the age of ≥ 16 y), and (4) elevated blood pressure (≥ 130/85 mmHg). In this method, those with one or no risk factor were considered as MHO adolescents. In the second classification, we added insulin resistance, as defined by the HOMA-IR score, to the IDF criteria, which was applied in the first classification^32^. Thus, students with HOMA-IR score ≥ 3.16 and two or more metabolic risk factors were considered as MUO individuals and those with HOMA-IR < 3.16 were considered as MHO. The cut-off value of 3.16 for HOMA-IR was selected on the basis of several prior studies among children and adolescents^33–35^.

### Assessment of other variables

Physical activity level of each participant was evaluated by the Physical Activity Questionnaire for Adolescents (PAQ-A) questionnaire, which includes nine questions on various activities during weekdays and weekend days^36^. Items 1–8 of this questionnaire are about the usual activity of adolescents and the last item is about unusual activity of adolescents during the last week. On the basis of total scores, adolescents were classified as active (score ≥ 3), low active (3 < score ≤ 2), sedentary (or not having a regular week activity) (score < 2). A trained interviewer administered a validated demographic questionnaire to evaluate socioeconomic status (SES) of individuals^37^, according to the following variables: job of parents, size of family, education of parents, taking trips, and having cars and laptops/computers for the family, and having private room for the student. Then, a total score was calculated for SES. Furthermore, information of age, sex, history of diseases, and use of medications and dietary supplements of participants was gathered through a questionnaire.

### Statistical analysis

Factor analysis with orthogonal transformation (varimax procedure) was applied to derive nutrient patterns based on 34 nutrients and bioactive compounds. Factors were retained for further analysis based on their natural interpretation, eigenvalues, and Scree plot^38^. In this study, we retained factors with eigenvalues > 2, as this cutoff could result in more interpretable nutrient patterns. Factor loadings for each nutrient were calculated and factor scores for each NP were obtained by summing up the total grams of all nutrients weighted by their factor loadings^38^. Each participant received a factor score for each identified NP^23^. NPs were labeled based on the nutrient groups loading high in each pattern. Then, participants were categorized into tertiles of NP scores. One-way ANOVA and chi-square tests were used to examine the differences in quantitative and categorical variables across tertiles of major NPs. Binary logistic regression was used to have OR and 95% CI for MUO across tertiles of major NPs. Age, sex, and energy intake were adjusted in the first model. Additional adjustments were done for physical activity, and socioeconomic status in the second model. In the last model, further adjustment for BMI was added to determine an independent relation from obesity. Participants in the first tertile of major NPs were considered as the reference category in all models. Tertiles of each NP were treated as an ordinal variable in order to determine P for trend. SPSS version 20 was applied to conduct all statistical analyses. P values were considered significant at < 0.05.

### Ethical approval and consent to participate

The study procedure was performed according to declaration of Helsinki and STROBE checklist. All participants provided informed written consent. The study protocol was approved by the local Ethics Committee of Isfahan University of Medical Sciences. Informed consent was obtained from all participants involved in the study.

## Results

Three major nutrient patterns (NPs) were identified among our participants (Table 1). Factor 1 (NP1), labeled as "high minerals and vitamins", contained a high intake of potassium, magnesium, folate, pantothenic acid, riboflavin, phosphorus, zinc, calcium, vitamin B12, B6 and C, and fiber. Factor 2 (NP2), labeled as "high carbohydrate", had highly intake of thiamin, niacin, carbohydrate, and iron. Factor 3 (NP3), labeled as "high fat and sodium", was characterized by high intake of poly-unsaturated fatty acids (PUFAs), sodium, saturated fatty acids (SFAs), and mono unsaturated fatty acids (MUFAs). These 3 factors explained 69% of total variance of nutrient intake. The KMO coefficient was 0.85, indicating adequate sampling.

**Table 1:** Factor loadings and explained variances for major nutrient patterns (NPs).

	Factor loadings		
	NP1 High minerals and vitamins	NP2 High carbohydrate	NP3 High fat and sodium
Potassium (mg/d)	0.948	–	–
Magnesium (mg/d)	0.924	–	0.288
Folate (mcg/d)	0.900	–	–
Pantothenic acid (mg/d)	0.882	–	0.296
Riboflavin (mg/d)	0.876	–	0.306
Phosphorus (mg/d)	0.864	–	0.376
Zinc (mg/d)	0.852	–	0.361
Calcium (mg/d)	0.851	–	–
Cobalamin (mg/d)	0.826	–	0.214
Pyridoxine (mg/d)	0.814	0.269	0.256
Vitamin C (mg/d)	0.811	–	–
Total fiber (g/d)	0.804	0.401	–
Vitamin K (mcg/d)	0.763	–	–
Sugar (g/d)	0.758	–	–
Protein (g/d)	0.719	0.489	0.296
Vitamin A (RE/d)	0.665	–	–
Copper (mg/d)	0.650	0.321	0.486
Biotin (mcg/d)	0.649	0.391	0.299
Cholesterol (mg/d)	0.608	–	0.300
Manganese (mg/d)	0.561	–	0.482
Vitamin D (mcg/d)	0.441	–	0.411
Thiamin (mg/d)	0.270	0.896	–
Niacin (mg/d)	0.275	0.892	0.207
Carbohydrate (g/d)	0.351	0.852	–
Iron (mg/d)	–	0.829	–
Chromium (mg/d)	–	0.567	0.455
Selenium (mg/d)	–	0.473	0.416
PUFA (g/d)	–	–	0.712
Sodium (mg/d)	0.229	0.314	0.647
SFA (g/d)	0.607	–	0.617
MUFA (g/d)	0.544	–	0.609
Vitamin E (mg/d)	–	–	0.581
TFA (g/d)	–	0.296	0.548
Fluoride (mcg/d)	0.215	− 0.243	0.324
Variance explained (%)	40.92	14.38	13.62
Cumulative explained variance (%)	40.92	55.30	68.92

Values are factor loadings. Factor loadings < │0.20│ are not shown for simplicity. The Kaiser–Meyer–Olkin value was 0.85. Factors with Eigen values of ≥ 2 were retained to extract major NPs.

Table 2 indicates general features of participants across tertiles of major NPs. In comparison to the lowest tertile of high minerals and vitamins pattern, participants in the highest tertile were more likely to be boy, have lower physical activity and socioeconomic status, higher HDL-c, lower FBG and TG levels. In comparison to those in the lowest tertile of high carbohydrate pattern, adolescents in the highest tertile had higher weight, waist circumference (WC), SBP, DPB, and FBG, and were more likely to be boy. In terms of high fat and sodium pattern, participants in the top tertile were more likely to be boy, have higher weight, WC, FBG, TG, insulin, HOMA-IR index, and lower HDL-c and less likely to be physical active, compared to the bottom tertile.

**Table 2:** General characteristics and cardiometabolic factors of study participants across tertiles of major nutrient patterns.

	Tertiles of NP1 High minerals and vitamins			*P* value^a^	Tertiles of NP2 High carbohydrate			*P* value^a^	Tertiles of NP3 High fat and sodium			*P* value^a^
	T1 (n = 67)	T2 (n = 68)	T3 (n = 68)		T1 (n = 67)	T2 (n = 68)	T3 (n = 68)		T1 (n = 67)	T2 (n = 68)	T3 (n = 68)	
**Sex, n (%)**												
Boys	23 (34.3)	30(44.1)	48(70.6)	< 0.001	25 (37.3)	26 (38.2)	50 (73.5)	< 0.001	24 (35.8)	37(54.4)	40 (58.8)	0.01
Girls	44 (65.7)	38 (55.9)	20 (29.4)		42 (62.7)	42 (61.8)	18 (26.5)		43 (64.2)	31 (45.6)	28 (41.2)	
Age (year)	14.1 ± 1.64	13.9 ± 1.49	13.8 ± 1.69	0.44	14.0 ± 1.61	14.1 ± 1.78	13.8 ± 1.41	0.59	14.0 ± 1.62	13.9 ± 1.69	14.0 ± 1.53	0.88
Weight (kg)	73.5 ± 11.78	73.3 ± 11.63	73.7 ± 11.57	0.98	70.1 ± 9.68	72.8 ± 10.53	77.4 ± 13.23	0.01	69.7 ± 10.21	73.0 ± 10.44	77.7 ± 12.71	< 0.001
Height (cm)	162.8 ± 7.97	163.4 ± 7.49	164.8 ± 8.33	0.32	161.9 ± 8.14	162.7 ± 7.39	166.2 ± 7.73	0.03	161.2 ± 5.88	163.6 ± 7.35	166.1 ± 9.49	0.001
BMI (kg/m^2^)	27.6 ± 3.03	27.3 ± 3.06	27.1 ± 3.61	0.64	26.7 ± 2.63	27.4 ± 2.82	27.9 ± 4.01	0.08	26.8 ± 3.66	27.2 ± 2.86	28.0 ± 3.08	0.08
Waist circumference (cm)	90.2 ± 6.98	90.8 ± 7.6	89.9 ± 9.15	0.83	88.8 ± 7.60	89.7 ± 6.73	92.4 ± 8.97	0.02	87.3 ± 6.57	89.9 ± 7.19	93.8 ± 8.63	< 0.001
**Physical activity levels, n (%)**												
Sedentary	46 (68.7)	27 (39.7)	16 (23.5)	< 0.001	26 (38.8)	27 (39.7)	36 (52.9)	0.34	16 (23.9)	34 (50.0)	39 (57.4)	< 0.001
Low-activity	19 (28.4)	28 (41.2)	30 (44.1)		26 (38.8)	27 (39.7)	24 (35.3)		28 (41.8)	36 (32.4)	27 (39.7)	
Active	2 (3.0)	13 (19.1)	22 (32.4)		15 (22.4)	14 (20.6)	8 (11.8)		23 (34.3)	10 (17.6)	2 (2.9)	
**Socioeconomic status**^**b**^**, n (%)**												
Low	27 (40.3)	20 (29.4)	12 (17.6)	0.02	18 (26.9)	21 (30.9)	20 (29.4)	0.60	16 (23.9)	22 (32.4)	21 (30.9)	0.52
Medium	28 (41.8)	32 (47.1)	30 (44.1)		34 (50.7)	30 (44.1)	26 (38.2)		35 (52.2)	29 (42.6)	26 (38.2)	
High	12 (17.9)	16 (23.5)	26 (38.2)		15 (22.4)	17 (25.0)	22 (32.4)		16 (23.9)	17 (25.0)	21 (30.9)	
Systolic blood pressure (mmHg)	112.0 ± 16.88	112.8 ± 21.81	113.3 ± 16.06	0.93	109.3 ± 23.66	110.2 ± 16.69	118.5 ± 11.51	0.005	109.9 ± 17.54	111.6 ± 20.41	116.6 ± 16.44	0.08
Diastolic blood pressure (mmHg)	74.1 ± 13.21	73.4 ± 10.69	73.1 ± 10.17	0.87	70.8 ± 13.09	73.8 ± 10.46	75.8 ± 9.98	0.04	71.3 ± 12.83	73.9 ± 10.55	75.2 ± 10.42	0.13
Fasting blood glucose level (mg/dL)	100.3 ± 9.8	97.8 ± 7.76	96.4 ± 7.43	0.03	96.5 ± 7.75	97.7 ± 8.79	100.1 ± 8.66	0.04	93.8 ± 5.64	97.7 ± 8.13	102.7 ± 9.03	< 0.001
Insulin (μUI/mL)	22.2 ± 14.14	19.9 ± 11.69	19.2 ± 12.02	0.37	18.2 ± 11.81	19.8 ± 14.76	23.2 ± 10.73	0.07	15.2 ± 7.16	20.9 ± 11.08	25.0 ± 16.16	< 0.001
HOMA-IR index	5.48 ± 3.34	4.89 ± 3.09	4.69 ± 3.29	0.36	4.45 ± 3.22	4.82 ± 3.59	5.78 ± 2.88	0.05	3.55 ± 1.76	5.15 ± 3.01	6.34 ± 4.04	< 0.001
Triglycerides (mg/dL)	132.5 ± 71.40	129.2 ± 66.31	104.3 ± 58.77	0.03	109.9 ± 66.74	122.0 ± 64.96	133.7 ± 66.81	0.12	101.2 ± 50.29	124.6 ± 58.98	139.8 ± 81.39	0.003
HDL cholesterol (mg/dL)	43.4 ± 7.34	43.6 ± 7.69	47.5 ± 8.12	0.002	45.7 ± 8.55	45.2 ± 7.05	43.6 ± 8.07	0.28	46.8 ± 6.27	44.7 ± 9.58	42.9 ± 7.16	0.02

Values are Mean ± SD; unless indicated. *BMI* body mass index, *HOMA-IR* homeostasis model assessment insulin resistance, *HDL-c* high-density lipoprotein cholesterol, *n* number, *mmHg* millimeter.

^a^*P* value for independent two ANOVA and χ^2^ test for quantitative and categorical variables, respectively.

^b^Socioeconomic status (SES) score was evaluated based on parental education level, parental job, family size, having car in the family, having computer/laptop, having personal room and having travel by using a demographic questionnaire.

Dietary intakes of participants across tertiles of major NPs are provided in Table 3. Adolescents in the highest tertile of high minerals and vitamins pattern had higher intake of protein, SFA, vitamin C, pyridoxine, folate, calcium, fiber, vegetables, fruits, dairy products, whole grain, legumes, and nuts and lower consumption of carbohydrates, vitamin E, iron, refined grain, and linoleic acid (LA) than adolescents in the lowest category. In case of high carbohydrate pattern, adolescents in the top category had greater intake of energy, iron, carbohydrate, and refined grain and lower consumption of proteins, fat, SFA, vitamin C, pyridoxin, E, folate, calcium, vegetables, fruits, dairies, legumes, nuts, omega-6, and LA than those in the first category. Compared with adolescents in the lowest tertile of high fat and sodium pattern, those in the highest tertile had higher consumption of fat, energy, sodium, vitamin E, omega-3, and LA and lower intake of protein, carbohydrate, vitamin C, folate, iron, fiber, vegetables, fruits, and whole grains.

**Table 3:** Dietary intakes of study participants across tertiles of major nutrient patterns (NPs).

	Tertiles of NP1 High minerals and vitamins				Tertiles of NP2 High carbohydrate				Tertiles of NP3 High fat and sodium			
	T1 (n = 67)	T2 (n = 68)	T3 (n = 68)	*P* value^a^	T1 (n = 67)	T2 (n = 68)	T3 (n = 68)	*P* value^a^	T1 (n = 62)	T2 (n = 74)	T3 (n = 67)	*P* value^a^
Energy, kcal	2864.2 ± 67.05	2800.9 ± 65.59	2983.4 ± 67.53	0.16	2677.3 ± 62.81	2802.9 ± 62.16	3165.9 ± 64.26	< 0.001	2802.1 ± 65.50	2778.8 ± 64.19	3066.9 ± 64.56	0.003
Protein, % of E	12.9 ± 0.21	14.2 ± 0.21	15.8 ± 0.21	< 0.001	15.2 ± 0.25	14.2 ± 0.24	13.6 ± 0.26	< 0.001	14.9 ± 0.25	14.1 ± 0.24	13.8 ± 0.25	0.005
Carbohydrate, % of E	60.1 ± 0.61	58.9 ± 0.60	55.9 ± 0.62	< 0.001	53.5 ± 0.50	58.6 ± 0.48	62.7 ± 0.52	< 0.001	61.9 ± 0.55	58.2 ± 0.54	54.8 ± 0.55	< 0.001
Fat, % of E	27.9 ± 0.64	28.4 ± 0.63	30.2 ± 0.64	0.03	33.2 ± 0.53	28.6 ± 0.51	24.8 ± 0.55	< 0.001	24.7 ± 0.51	29.1 ± 0.50	32.7 ± 0.51	< 0.001
SFA, gr	24.7 ± 0.67	27.1 ± 0.66	30.3 ± 0.68	< 0.001	32.2 ± 0.56	27.4 ± 0.57	22.6 ± 0.61	< 0.001	24.5 ± 0.65	26.8 ± 0.64	30.8 ± 0.65	< 0.001
Vitamin C, mg	84.8 ± 8.13	136.2 ± 5.59	179.2 ± 5.77	< 0.001	154.0 ± 7.26	134.0 ± 7.03	113.2 ± 7.58	0.001	161.4 ± 6.72	138.1 ± 6.60	101.7 ± 6.73	< 0.001
Vitamin B6, mg	1.29 ± 0.04	1.63 ± 0.04	1.94 ± 0.04	< 0.001	1.85 ± 0.05	1.64 ± 0.05	1.38 ± 0.05	< 0.001	1.72 ± 0.05	1.59 ± 0.05	1.56 ± 0.05	0.07
Vitamin E, mg	34.1 ± 1.39	30.4 ± 1.37	26.6 ± 1.41	0.001	32.9 ± 1.44	31.1 ± 1.39	27.0 ± 1.51	0.03	24.2 ± 1.30	30.4 ± 1.28	36.5 ± 1.31	< 0.001
Folate, mcg	228.4 ± 8.62	305.6 ± 8.46	414.5 ± 8.73	< 0.001	379.0 ± 11.39	319.2 ± 12.02	252.5 ± 11.89	< 0.001	344.7 ± 12.27	316.2 ± 12.05	289.4 ± 12.29	0.008
Iron, mg	27.3 ± 0.63	25.04 ± 0.62	22.5 ± 0.64	< 0.001	21.6 ± 0.58	23.9 ± 0.56	29.2 ± 0.61	< 0.001	27.0 ± 0.63	25.5 ± 0.62	22.3 ± 0.63	< 0.001
Calcium, mg	1047.9 ± 30.6	1298.5 ± 30.0	1661 ± 30.9	< 0.001	1573.7 ± 37.56	1352.9 ± 36.35	1089.1 ± 39.21	< 0.001	1419.7 ± 42.36	1308.8 ± 41.63	1285.0 ± 42.43	0.06
Total fiber, gr	15.6 ± 0.48	19.6 ± 0.47	23.1 ± 0.49	< 0.001	20.2 ± 0.61	19.8 ± 0.59	18.3 ± 0.64	0.113	22.5 ± 0.51	19.9 ± 0.50	16.0 ± 0.51	< 0.001
Sodium, mg	4189.4 ± 141.54	4942.9 ± 138.99	3736.8 ± 143.33	0.09	4326.9 ± 143.30	3958.9 ± 138.71	3685.8 ± 149.60	0.01	3334.2 ± 129.15	4028.7 ± 126.91	4593.7 ± 129.38	< 0.001
**Food groups**												
Vegetables, g/d	172.9 ± 18.09	263.7 ± 17.76	419.1 ± 18.31	< 0.001	316.0 ± 21.85	309.3 ± 21.15	232.6 ± 22.81	0.02	330.8 ± 20.94	311.4 ± 20.57	215.9 ± 20.97	< 0.001
Fruits, g/d	195.7 ± 16.71	332.1 ± 16.41	405.0 ± 16.9	< 0.001	358.4 ± 19.67	312.5 ± 19.04	264.3 ± 20.53	0.008	400.6 ± 17.78	307.9 ± 17.47	227.4 ± 17.8	< 0.001
Dairy, g/d	344.1 ± 20.68	492.8 ± 20.31	707.2 ± 20.94	< 0.001	678.4 ± 23.23	522.2 ± 22.49	348.3 ± 24.26	< 0.001	523.7 ± 27.26	494.9 ± 26.79	528.0 ± 27.31	0.64
Whole grains, g/d	57.9 ± 12.30	87.1 ± 12.08	119.5 ± 12.46	0.03	79.8 ± 12.84	106.6 ± 12.43	78.5 ± 13.41	0.20	144.2 ± 11.68	72.2 ± 11.48	49.5 ± 11.70	< 0.001
Refined grain, g/d	693.2 ± 16.56	541.1 ± 16.26	409.6 ± 16.77	< 0.001	411.3 ± 18.22	551.5 ± 17.64	676.9 ± 19.02	< 0.001	506.8 ± 21.35	573.5 ± 20.98	560.8 ± 21.38	0.07
Legume, g/d	33.9 ± 3.32	49.3 ± 3.26	56.4 ± 3.36	< 0.001	53.6 ± 3.49	49.0 ± 3.39	37.2 ± 3.37	0.008	53.2 ± 3.45	46.1 ± 3.39	40.4 ± 3.46	0.04
Nuts, g/d	7.96 ± 1.26	10.25 ± 1.24	18.261.28	< 0.001	16.15 ± 1.35	12.27 ± 1.31	8.17 ± 1.41	0.001	12.2 ± 1.37	13.0 ± 1.34	11.3 ± 1.37	0.66
Meat, g/d	64.87 ± 4.07	70.2 ± 3.99	70.94 ± 4.12	0.53	71.8 ± 4.16	69.0 ± 4.03	65.2 ± 4.35	0.58	61.5 ± 4.03	69.5 ± 3.96	74.9 ± 4.04	0.07
Omega-3, g/d	0.61 ± 0.02	0.61 ± 0.02	0.59 ± 0.02	0.85	0.67 ± 0.02	0.59 ± 0.02	0.55 ± 0.22	< 0.001	0.50 ± 0.02	0.61 ± 0.02	0.69 ± 0.02	< 0.001
Linoleic acids, g/d	28.42 ± 0.98	24.92 ± 0.96	23.42 ± 0.99	0.002	28.41 ± 0.99	25.79 ± 0.96	22.56 ± 1.04	0.001	19.51 ± 0.83	25.94	31.17 ± 0.83	< 0.001

Values are Mean ± SE. Energy intake and macronutrients were adjusted for age and sex; all other values were adjusted for age, sex and energy intake. *E* energy intake, *SFA* saturated fatty acids, *MUFA* monounsaturated fatty acids, *PUFA* polyunsaturated fatty acids, *LA* linoleic acid.

^a^*P* value obtained from ANCOVA test.

**Figure 1:**
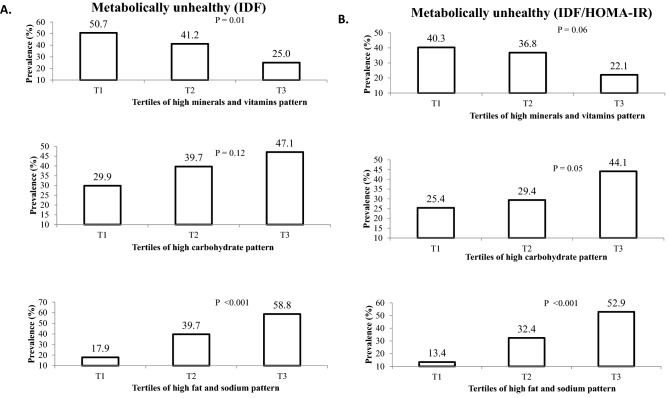
Prevalence of MUO across tertiles of major nutrient patterns (NPs). (**A**) Based on IDF definition, (**B**) Based on IDF/HOMA-IR definition.

The prevalence of MUO across tertiles of different nutrient patterns among study population is shown in Fig. 1. Based on IDF definition, the prevalence of MUO in the top tertile of NP1 was significantly lower in comparison to the bottom tertile (25.0 vs. 50.7%, *P* = 0.01). MUO prevalence among individuals in the highest category of NP2 was not significantly different from the lowest tertile (47.1 vs. 29.9%, *P* = 0.12). On the other hand, prevalence of MUO in the last category of NP3 was higher than the first category (58.8 vs. 17.9%, *P* < 0.001). According to the second definition of metabolic health status (IDF/HOMA-IR), the prevalence of MUO in the highest tertile of NP1 was marginally significantly different from the lowest tertile (22.1 vs. 40.3%, *P* = 0.06). Adolescents in the top tertile of NP2 compared to those in the bottom tertile, had slightly higher prevalence of MUO (44.1 vs. 25.4%, *P* = 0.05). The prevalence of MUO was also significantly higher in the third tertile of NP3 in comparison to the first one (52.9 vs. 13.4%, *P* < 0.001).

Crude and multivariable-adjusted ORs and 95% confidence intervals (CIs) for MUO across tertiles of major NPs are provided in Table 4. Based on IDF definition, a significant inverse relation was observed between adherence to the NP1 (high minerals and vitamins) and MUO (OR = 0.32; 95% CI 0.16, 0.52) in crude model. This relation remained significant after controlling for age, sex and energy intake (OR = 0.24; 95% CI 0.10, 0.54). However, after considering other potential confounders, this association became non-significant. Greater adherence of NP2 (high carbohydrate) was positively associated with MUO (OR = 2.09, 95% CI 1.03, 4.24) in crude model; however, no significant association was observed after more controlling for other confounding variables. Compared to adolescents in the lowest tertile of NP3 (high fat and sodium), those with the highest adherence to this pattern were more likely to be MUO (OR = 6.55; 95% CI 2.97, 14.42), in crude model. This association was significant even after adjustment for all potential confounders; such that, adolescents in the top category of high fat and sodium pattern compared with the bottom category had a 212% increased odds for MUO (OR = 3.12; 95% CI 1.19, 8.09), in the fully-adjusted model.

**Table 4:** Multivariate adjusted odds ratio (OR) and 95% confidence interval (CI) for MUO phenotype across tertiles of major nutrient patterns (NPs).

	Tertiles of NP1 High minerals and vitamins				Tertiles of NP2 High carbohydrate				Tertiles of NP3 High fat and sodium			
	T1 (n = 67)	T2 (n = 68)	T3 (n = 68)	P-trend^a^	T1 (n = 67)	T2 (n = 68)	T3 (n = 68)	P-trend^a^	T1 (n = 67)	T2 (n = 68)	T3 (n = 68)	P-trend^a^
**MUO phenotype based on IDF criteria**												
Cases (n)	34	28	17		20	27	32		12	27	40	
Crude	1 (Ref.)	0.68 (0.34, 1.34)	0.32 (0.16, 0.52)	0.002	1 (Ref.)	1.55 (0.76, 3.16)	2.09 (1.03, 4.24)	0.04	1 (Ref.)	3.02 (1.37, 6.66)	6.55 (2.97, 14.42)	< 0.001
Model 1	1 (Ref.)	0.74 (0.36, 1.50)	0.24 (0.10, 0.54)	0.001	1 (Ref.)	1.31 (0.62, 2.74)	1.29 (0.55, 3.04)	0.53	1 (Ref.)	3.53 (1.55, 8.06)	6.06 (2.62, 14.03)	< 0.001
Model 2	1 (Ref.)	1.32 (0.58, 2.98)	0.53 (0.21, 2.98)	0.29	1 (Ref.)	1.52 (0.65, 3.55)	0.91 (0.35, 2.36)	0.89	1 (Ref.)	2.11 (0.84, 5.27)	3.53 (1.39, 9.00)	0.01
Model 3	1 (Ref.)	1.31 (0.57, 2.99)	0.55 (0.22, 1.41)	0.32	1 (Ref.)	1.31 (0.55, 3.12)	0.92 (0.35, 2.40)	0.89	1 (Ref.)	1.84 (0.72, 4.69)	3.12 (1.19, 8.09)	0.03
**MUO phenotype based on HOMA-IR criteria**												
Cases (n)	27	25	15		17	20	30		9	22	36	
Crude	1 (Ref.)	0.86 (0.43, 1.72)	0.42 (0.19, 0.89)	0.03	1 (Ref.)	1.23 (0.57, 2.62)	2.32 (1.12, 4.82)	0.02	1 (Ref.)	3.08 (1.29, 7.33)	7.25 (3.10, 16.94)	< 0.001
Model 1	1 (Ref.)	0.93 (0.44, 1.94)	0.26 (0.11, 0.61)	0.003	1 (Ref.)	0.99 (0.45, 2.19)	1.14 (0.47, 2.19)	0.78	1 (Ref.)	3.44 (1.39, 8.48)	5.89 (2.41, 14.40)	< 0.001
Model 2	1 (Ref.)	1.69 (0.72, 3.98)	0.58 (0.22, 1.55)	0.46	1 (Ref.)	1.09 (0.44, 2.66)	0.79 (0.29, 1.10)	0.62	1 (Ref.)	1.94 (0.72, 5.26)	3.35 (1.25, 8.97)	0.02
Model 3	1 (Ref.)	1.65 (0.69, 3.94)	0.59 (0.22, 1.59)	0.47	1 (Ref.)	0.96 (0.42, 2.21)	0.46 (0.16, 1.29)	0.64	1 (Ref.)	1.57 (0.56, 4.40)	2.81 (1.02, 7.74)	0.05

All values are odds ratios and 95% confidence intervals. Model 1: Adjusted for age, sex, and energy intake. Model 2: Additionally, adjusted for physical activity and socioeconomic status (evaluated based on parental education level, parental job, family size, having car in the family, having computer/laptop, having personal room and having travel by using demographic questionnaire). Model 3: More adjustments were done for body mass index (BMI).

^a^Obtained by the use of tertiles of major nutrient patterns as an ordinal variable in the model.

According to the IDF/HOMA-IR definition, a significant inverse association between NP1 and MUO was observed (OR = 0.42; 95% CI 0.19, 0.89), in crude model. This association strengthened after controlling for age, sex, and energy intake (OR = 0.26; 95% CI 0.11, 0.61). However, this association disappeared after further adjustments for other confounders. Adolescents with the highest adherence to NP2 were 2.32 times more likely to be MUO in crude model (OR = 2.32; 95% CI 1.12, 4.82); but this association became non-significant, after making more adjustments. Individuals in the highest tertile of NP3 had a significant increased odds of MUO both before (OR = 7.25; 95% CI 3.10, 16.94) and after adjustments for all potential confounders (OR = 2.81; 95% CI 1.02, 7.74); such that, adolescents in the top tertile of NP3 had a 181% higher odds of MUO in fully-adjusted model, compared to those in the lowest tertile.

As shown in Table 5, stratified analysis by sex revealed that after controlling for age and energy intake, girls in the highest vs. lowest tertile of NP1 were respectively 92% and 88% less likely to be MUO based on IDF (OR = 0.12; 95% CI 0.03, 0.52) and IDF/HOMA-IR definition (OR = 0.08; 95% CI 0.01, 0.50). These relations became non-significant after further adjustments. Based on IDF definition, among boys, a significant lower odds of being MUO was seen in the top vs. bottom category of NP1 (OR = 0.31; 95% CI 0.11, 0.37) in the crude model. A significant direct relation between NP2 and MUO based on IDF (OR = 4.46; 95% CI 1.37, 14.49) and IDF/HOMA-IR definition (OR = 5.03; 95% CI 1.15, 16.43) was observed in girls, in crude model; however, this association disappeared in fully-adjusted model. Among girls and boys, higher adherence to NP3 was associated with greater odds of being MUO in crude model and after adjustments for age and energy (based on IDF definition, model 1, for girls: OR = 6.34; 95% CI 2.01, 19.97; for boys: OR = 7.15; 95% CI 1.74, 29.39; based on IDF/HOMA-R definition, model 1, for girls: OR = 8.22; 95% CI 2.62, 25.76; for boys: OR = 7.00; 95% CI 1.79, 27.25). These associations disappeared in fully-adjusted model.

**Table 5:** Multivariate adjusted odds ratio (OR) and 95% confidence interval (CI) for MUO phenotype across tertiles of major nutrient patterns (NPs), stratified by sex.

	Tertiles of NP1 High minerals and vitamins				Tertiles of NP2 High carbohydrate				Tertiles of NP3 High fat and sodium			
	T1	T2	T3	P-trend^a^	T1	T2	T3	P-trend^a^	T1	T2	T3	P-trend^a^
**MUO phenotype based on IDF criteria**												
**Girls (Participants/Cases)**	44/22	38/15	20/5		42/13	42/17	18/12		43/9	31/14	28/19	
Crude	1 (Ref.)	0.65 (0.27, 1.57)	0.33 (0.10, 1.08)	0.06	1 (Ref.)	1.52 (0.62, 3.73)	4.46 (1.37, 14.49)	0.02	1 (Ref.)	3.11 (1.12, 8.63)	7.98 (2.71, 23.51)	< 0.001
Model 1	1 (Ref.)	0.69 (0.26, 1.82)	0.12 (0.03, 0.52)	0.007	1 (Ref.)	1.15 (0.45, 2.98)	2.10 (0.56, 7.89)	0.32	1 (Ref.)	2.86 (0.99, 8.24)	6.34 (2.01, 19.97)	0.001
Model 2	1 (Ref.)	1.32 (0.41, 4.22)	0.27 (0.05, 1.51)	0.38	1 (Ref.)	1.17 (0.38, 3.54)	1.36 (0.32, 5.33)	0.79	1 (Ref.)	1.53 (0.45, 5.17)	3.15 (0.85, 11.61)	0.09
Model 3	1 (Ref.)	1.32 (0.41, 4.23)	0.28 (0.05, 1.60)	0.41	1 (Ref.)	1.09 (0.35, 3.44)	1.33 (0.31, 5.73)	0.79	1 (Ref.)	1.47 (0.42, 5.12)	3.06 (0.82, 11.41)	0.10
**Boys** **(Participants/Cases)**	23/12	30/13	48/12		25/7	26/10	50/20		24/3	37/13	40/21	
Crude	1 (Ref.)	0.70 (0.24, 2.09)	0.31 (0.11, 0.87)	0.02	1 (Ref.)	1.61 (0.49, 5.22)	1.71 (0.61, 4.85)	0.34	1 (Ref.)	3.79 (0.95, 15.15)	7.74 (1.99, 30.13)	0.002
Model 1	1 (Ref.)	0.75 (0.24, 2.29)	0.26 (0.09, 0.79)	0.01	1 (Ref.)	1.44 (0.43, 4.80)	0.97 (0.30, 3.12)	0.88	1 (Ref.)	4.63 (1.10, 19.44)	7.15 (1.74, 29.39)	0.006
Model 2	1 (Ref.)	1.26 (0.35, 4.58)	0.56 (0.16, 1.97)	0.31	1 (Ref.)	2.21 (0.54, 8.98)	0.79 (0.21, 2.96)	0.67	1 (Ref.)	3.04 (0.64, 14.36)	4.84 (1.02, 22.96)	0.07
Model 3	1 (Ref.)	1.07 (0.29, 3.99)	0.46 (0.12, 1.75)	0.24	1 (Ref.)	1.96 (0.45, 8.54)	0.88 (0.22, 3.51)	0.81	1 (Ref.)	2.54 (0.52, 12.48)	4.20 (0.83, 21.14)	0.12
**MUO phenotype based on HOMA-IR criteria**												
**Girls** **(Participants/Cases)**	44/16	38/13	20/3		42/10	42/11	18/11		43/6	31/10	28/16	
Crude	1 (Ref.)	0.91 (0.37, 2.26)	031 (0.08, 1.22)	0.13	1 (Ref.)	1.14 (0.42, 3.05)	5.03 (1.54, 16.43)	0.02	1 (Ref.)	2.94 (0.93, 9.23)	8.22 (2.62, 25.76)	< 0.001
Model 1	1 (Ref.)	1.09 (0.38, 3.11)	0.08 (0.01, 0.50)	0.02	1 (Ref.)	0.84 (0.29, 2.42)	2.16 (0.56, 8.37)	0.37	1 (Ref.)	2.69 (0.81, 8.96)	5.76 (1.70, 19.46)	0.005
Model 2	1 (Ref.)	2.53 (0.69, 9.34)	0.21 (0.03, 1.69)	0.67	1 (Ref.)	0.71 (0.20, 2.46)	1.32 (0.28, 6.21)	0.83	1 (Ref.)	1.11 (0.27, 4.58)	2.37 (0.59, 9.59)	0.19
Model 3	1 (Ref.)	2.53 (0.68, 9.39)	0.25 (0.03, 2.06)	0.76	1 (Ref.)	0.52 (0.14, 1.97)	1.21 (0.25, 5.86)	0.94	1 (Ref.)	0.86 (0.19, 3.87)	2.11 (0.51, 8.80)	0.23
**Boys** **(Participants/Cases)**	23/11	30/12	48/12		25/7	26/9	50/19		24/3	37/12	40/20	
Crude	1 (Ref.)	0.73 (0.24, 2.18)	0.36 (0.13, 1.03)	0.05	1 (Ref.)	1.36 (0.41, 4.47)	1.58 (0.56, 4.47)	0.039	1 (Ref.)	3.36 (0.84, 13.52)	7.00 (1.79, 27.25)	0.003
Model 1	1 (Ref.)	0.79 (0.26, 2.49)	0.32 (0.10, 0.97)	0.04	1 (Ref.)	1.19 (0.35, 4.06)	0.80 (0.25, 2.61)	0.65	1 (Ref.)	4.19 (0.98, 17.93)	6.19 (1.49, 25.64)	0.01
Model 2	1 (Ref.)	1.38 (0.38, 5.07)	0.69 (0.19, 2.49)	0.53	1 (Ref.)	1.75 (0.42, 7.26)	0.63 (0.16, 2.39)	0.46	1 (Ref.)	2.82 (0.59, 13.55)	4.41 (0.92, 21.26)	0.11
Model 3	1 (Ref.)	1.21 (0.33, 4.52)	0.60 (0.16, 2.28)	0.45	1 (Ref.)	1.57 (0.36, 6.81)	0.69 (0.17, 2.76)	0.58	1 (Ref.)	2.41 (0.48, 11.99)	3.79 (0.75, 19.20)	0.18

All values are odds ratios and 95% confidence intervals. Model 1: Adjusted for age and energy intake. Model 2: Additionally, adjusted for physical activity and socioeconomic status (evaluated based on parental education level, parental job, family size, having car in the family, having computer/laptop, having personal room and having travel by using demographic questionnaire). Model 3: More adjustments were done for body mass index (BMI).

^a^Obtained by the use of tertiles of major nutrient patterns as an ordinal variable in the model.

As shown in Table 6, stratified by BMI categories revealed that both overweight and obese adolescents in higher tertile of NP1 were less likely to be MUO based on both metabolic health criteria, but this association was only statistically significant in overweight adolescents in crude and the first model. According to IDF and HOMA-IR definitions for MUO, in crude and model 1, higher adherence to NP3 was associated with higher odds of MUO both in overweight and obese adolescents. However, in fully-adjusted model, the relation was significant only in obese adolescents (OR = 5.04; 95% CI 1.17, 21.78), based on IDF criteria.

**Table 6:** Multivariate adjusted odds ratio (OR) and 95% confidence interval (CI) for MUO phenotype across tertiles of major nutrient patterns (NPs), stratified by BMI categories.

	Tertiles of NP1 High minerals and vitamins				Tertiles of NP2 High carbohydrate				Tertiles of NP3 High fat and sodium			
	T1	T2	T3	P-trend^a^	T1	T2	T3	P-trend^a^	T1	T2	T3	P-trend^a^
**MUO phenotype based on IDF criteria**												
**Overweight**												
**(Participants/Cases)**	32/16	36/7	36/5		44/8	30/9	30/11		47/8	31/8	26/12	
Crude	1 (Ref.)	0.24 (0.82, 0.71)	0.16 (0.05, 0.52)	0.002	1 (Ref.)	1.93 (0.65, 5.76)	2.61 (0.89, 7.57)	0.08	1 (Ref.)	1.69 (0.56, 5.13)	4.18 (1.42, 12.34)	0.01
Model 1	1 (Ref.)	0.31 (0.09, 0.97)	0.10 (0.03, 0.42)	0.001	1 (Ref.)	1.79 (0.57, 5.61)	3.55 (0.92, 13.65)	0.07	1 (Ref.)	2.06 (0.63, 6.73)	4.71 (1.45, 15.34)	0.01
Model 2	1 (Ref.)	0.63 (0.17, 2.38)	0.30 (0.07, 2.18)	0.24	1 (Ref.)	2.66 (0.63, 11.13)	1.68 (0.35, 8.05)	0.40	1 (Ref.)	1.21 (0.31, 4.68)	2.07 (0.49, 8.59)	0.35
**Obese**												
**(Participants/Cases)**	35/18	32/21	32/12		23/12	38/18	38/21		20/4	37/19	42/28	
Crude	1 (Ref.)	1.80 (0.67, 4.83)	0.56 (0.21, 1.90)	0.77	1 (Ref.)	0.83 (0.29, 2.33)	1.13 (0.40, 3.19)	0.74	1 (Ref.)	4.22 (1.18, 15.05)	8.00 (2.25, 28.48)	0.001
Model 1	1 (Ref.)	1.66 (0.59, 4.63)	0.44 (0.14, 1.34)	0.18	1 (Ref.)	0.69 (0.24, 2.06)	0.56 (0.16, 1.97)	0.37	1 (Ref.)	4.75 (1.28, 17.58)	7.13 (1.91, 26.57)	0.004
Model 2	1 (Ref.)	2.67 (0.79, 8.94)	0.65 (0.18, 2.44)	0.63	1 (Ref.)	0.79 (0.24, 2.69)	0.56 (0.14, 2.24)	0.46	1 (Ref.)	3.04 (0.72, 12.81)	5.04 (1.17, 21.78)	0.05
**MUO phenotype based on HOMA-IR criteria**												
**Overweight**												
**(Participants/Cases)**	32/11	36/6	36/3		44/5	30/5	30/10		47/5	31/5	26/10	
Crude	1 (Ref.)	0.38 (0.12, 1.19)	0.17 (0.04, 0.69)	0.01	1 (Ref.)	1.56 (0.41, 5.94)	3.90 (1.17, 12.97)	0.03	1 (Ref.)	1.62 (0.43, 6.12)	5.25 (1.55, 17.75)	0.008
Model 1	1 (Ref.)	0.59 (0.17, 2.03)	0.08 (0.01, 0.43)	0.002	1 (Ref.)	1.34 (0.34, 5.24)	3.18 (0.77, 13.24)	0.12	1 (Ref.)	1.85 (0.46, 7.53)	4.80 (1.32, 17.49)	0.02
Model 2	1 (Ref.)	1.45 (0.33, 6.35)	0.38 (0.05, 2.89)	0.52	1 (Ref.)	1.89 (0.48, 7.35)	0.94 (0.12, 7.53)	0.70	1 (Ref.)	0.91 (0.18, 4.63)	1.84 (0.42, 8.04)	0.22
**Obese**												
**(Participants/Cases)**	35/16	32/19	32/12		23/12	38/15	38/20		20/4	37/17	42/26	
Crude	1 (Ref.)	1.74 (0.66, 4.58)	0.71 (0.27, 1.89)	0.53	1 (Ref.)	0.59 (0.21, 1.70)	1.02 (0.36, 2.87)	0.82	1 (Ref.)	3.40 (0.95, 12.13)	6.50 (1.84, 22.92)	0.003
Model 1	1 (Ref.)	1.59 (0.58, 4.39)	0.54 (0.18, 1.68)	0.34	1 (Ref.)	0.49 (0.16, 1.48)	0.46 (0.13, 1.66)	0.24	1 (Ref.)	3.81 (1.02, 14.18)	4.80 (1.32, 17.49)	0.01
Model 2	1 (Ref.)	2.29 (0.71, 7.40)	0.75 (0.19, 2.80)	0.89	1 (Ref.)	0.55 (0.16, 1.87)	0.46 (0.12, 1.87)	0.29	1 (Ref.)	2.44 (0.57, 10.41)	4.09 (0.94, 17.82)	0.25

All values are odds ratios and 95% confidence intervals. Model 1: Adjusted for age, sex, and energy intake. Model 2: Additionally, adjusted for physical activity and socioeconomic status (evaluated based on parental education level, parental job, family size, having car in the family, having computer/laptop, having personal room and having travel by using demographic questionnaire).

^a^Obtained by the use of tertiles of major nutrient patterns as an ordinal variable in the model.

## Discussion

The current investigation indicated that an empirically-derived pattern of high fat and sodium was positively associated with MUO in Iranian adolescents. This relation was independent from the criteria used to define metabolic health status. Findings from stratified analyses revealed that the observed associations were more considerably among boys and adolescents with obesity in comparison to girls and overweight individuals. To our knowledge, this is the first cross-sectional study that evaluated the link between nutrient patterns and metabolic health status among adolescents with overweight/obesity.

Most previous studies on the association of dietary intake and metabolic health status have focused on dietary patterns^39–41^ or a single nutrient intake^42,43^. NP analysis is a new approach in nutritional epidemiology that covers the consumption of all nutrients in addition to their interactions^23^. Thus, NPs rather than dietary patterns allowed us to more efficiently characterize and compare quality of dietary intake of study population^23^. In the current study, three major NPs with extremely complex nutritional profiles were extracted. NP1 included a high intake of potassium, magnesium, folate, pantothenic acid, riboflavin, phosphorous, zinc, calcium, cobalamin, pyridoxin, vitamin C, and total fiber. We labeled this NP "high minerals and vitamins". This NP seems to be a rich plant-based diet including high amounts of vegetables, whole grains, nuts, and legumes. However, due of its high cobalamin and phosphorous content, this pattern may contain some amount of animal-source foods, as well. In general, this NP was a healthy or prudent pattern and could decrease the odds of MUO among Iranian adolescents, although this relation became non-significant after taking potential confounders into account. NP2 labeled as "high carbohydrate" was rich in thiamin, niacin, carbohydrate, and iron. Very few nutrients were highly loaded in this pattern. Adolescents with highest adherence to this NP as compared to those with the lowest adherence (T3 vs. T1) had higher consumption of refined grains, carbohydrate, and energy intake, while consumption of whole grains was not significantly different among these categories. Moderate intake of TFA and dietary fiber in combination with high intake of thiamin, niacin, and carbohydrate, which especially came from refined grains, might result in a traditional nutrient pattern among these Iranian adolescents. A meta-analysis on 14 observational studies revealed that high consumption of refined grains was positively associated with MetS odds, whereas whole grain consumption was negatively associated with this syndrome^44^. In the present study, the interactions between metabolic disorder-inducing nutrients such as refined grain and TFA^45^ and metabolic disorder-protective nutrients such as thiamin, niacin and dietary fiber^46^ resulted in a non-significant relation between NP2 and MUO in the current study.

We documented a positive association between NP3 and MUO prevalence among adolescents in the current investigation. This pattern was characterized by high intake of PUFA, sodium, MUFA, and SFA. We labeled this NP as "high fat and sodium". Some nutrients in this pattern are found in animal-based diets, while some others are predominantly found in plant-based meals. Since nutrients in fruits and vegetables such as dietary fiber, vitamin C and K had low loadings in this NP, it could be a somehow western pattern. High intake of SFA and moderate intake of TFA in this NP has been positively linked to metabolic disorders in previous investigations^47^. In contrast, there were evidences indicating inverse associations between MUFA and omega-3 fatty acids with metabolic disorders^48,49^. The combination of metabolic disorder-protecting nutrients (MUFA and omega-3 fatty acids) and metabolic disorder-inducing nutrients (SFA, TFA, and omega-6 fatty acids) of NP3 made the interpretation of the relation of this pattern with MUO complicated. However, when examining the interactions among nutrients, the overall effect of this pattern raised the likelihood of MUO. A growing body of research indicates that most children consume insufficient omega-3 fatty acids^50^, and over the last three decades, as a result of modern agriculture, western diets have steadily decreased total fat and saturated fat intake. Also, consumption of omega-6 fatty acids has increased, while consumption of omega-3 fatty acids has dropped, resulting in a considerable increase in the omega-6/omega-3 fatty acid ratio. Elevated ratio of omega-6/omega-3 might also raise the risk of MUO among studied adolescents in the present investigation^51^. In the current study, the mean of sodium intake in boys was considerably higher than girls (4218 vs. 3762 mg/d). The range of sodium intake in boys was also wider than girls (2201–15,509 vs. 1580–8010 mg/d), which might somehow facilitate finding the association with outcome of interest in boys. In case fat intake, there was no significant difference between boys and girls (29.0 vs. 28.7% of total energy intake); however, the mean intake of TFAs and SFAs in boys was higher than girls [for TFAs: 6.5 vs. 5.4 g/d; and for SFAs: 30.6 vs. 24.2 g/d]. As a previous review has documented, TFAs and SFAs intake from various pre-packed foods and bakery products could enhance the risk of coronary heart disease, insulin resistance, MetS, and diabetes^52^.

Numerous pathways for the relation of nutrients with MUO status have been proposed. Endocannabinoids, lipids generated from omega-6, are regulated by two factors: (1) the ratio of omega-6 to omega-3 fatty acids intake; and (2) the activity of biosynthetic and catabolic enzymes that engaged in the pathways. These lipids play a critical role in appetite and metabolic regulation^53^ and increased endocannabinoid signaling can result in weight gain and an unhealthy metabolic profile^54^. SFA intake can additionally elevate serum postprandial non-esterified fatty acid (NEFA) levels and promotes insulin resistance^55^. In comparison to carbohydrate, MUFA, or even SFA consumption, TFA consumption had detrimental effects on insulin resistance indicators^56^. These unfavorable consequences include higher triglyceride levels^57^ postprandial insulin levels,^56^ and postprandial glucose levels, as well as reduced glucose absorption in skeletal and cardiac muscle^57^.

The current study has several advantages and disadvantages. First, a representative sample of Iranian adolescents with various socioeconomic levels was investigated. Second, two distinct methods were used to characterize metabolic health status. Third, several potential confounding variables have been taken into account in the analyses. However, some considerations should be made when interpreting our findings. The nature of our study was cross-sectional; therefore, we cannot confer a causal relationship between NPs and MUO in adolescents, because of the transposition of exposure and outcome. In addition, dietary intake was assessed through an FFQ, which might have resulted in misclassification of participants, despite the fact that this FFQ could appropriately predict the relationship between dietary intakes and various diseases in adolescents^28,29^. Moreover, recall bias and other potential reporting biases could have influenced the findings. Furthermore, data collection for dietary intakes was performed in an interview setting, which might lead to social desirability bias. Also, even after adjusting for several potential factors, residual confounders (such as sleep health, puberty status, and food habits) might affect the results.

In conclusion, this cross-sectional study highlighted that high adherence to a "high fat and sodium" nutrient pattern was related to elevated chance of being MUO in Iranian adolescents, especially in boys and obese individuals. Therefore, less consumption of sodium, TFAs, and SFAs could be recommended to adolescents especially boys with obesity to prevent MUO prevalence.

## Data Availability

The data that support the findings of this study are available from the corresponding author [PS], upon reasonable request.

## Abbreviations

FFQ: Food frequency questionnaire
OR: Odds ratios
95% CI: 95% Confidence interval
MHO: Metabolically healthy obese
BMI: Body mass index
HDL-c: High-density lipoprotein cholesterol
MUO: Metabolically unhealthy obese
IDF: International Diabetes Federation
PAQ-A: Physical Activity Questionnaire for Adolescents
HOMA-IR: Homeostasis Model Assessment Insulin Resistance
WHO: World Health Organization
SES: Socioeconomic status
PCA: Principal component analysis
SBP: Systolic blood pressure
DBP: Diastolic blood pressure
TG: Triglycerides
FBG: Fasting blood glucose
SPSS: Statistical package for the social sciences
ANOVA: Analysis of variance
SD: Standard deviation
SE: Standard error
WC: Waist circumference
GBD: Global burden of disease
PUFA: Poly unsaturated fatty acids
MUFA: Mono-unsaturated-fatty-acids
SFA: Saturated fatty acids
CMRF: Cardio-metabolic risk factors
MetS: Metabolic syndrome
TFAs: Trans fatty acids
SFAs: Saturated fatty acids

## References

[1] Abarca-Gómez L, Abdeen ZA, Hamid ZA, Abu-Rmeileh NM, Acosta-Cazares B, Acuin C (2017). Worldwide trends in body-mass index, underweight, overweight, and obesity from 1975 to 2016: A pooled analysis of 2416 population-based measurement studies in 128· 9 million children, adolescents, and adults. The Lancet.

[2] Dagpo TD, Nolan CJ, Delghingaro-Augusto V (2020). Exploring therapeutic targets to reverse or prevent the transition from metabolically healthy to unhealthy obesity. Cells.

[3] Lobstein T, Jackson-Leach R, Moodie ML, Hall KD, Gortmaker SL, Swinburn BA (2015). Child and adolescent obesity: Part of a bigger picture. The Lancet.

[4] Swinburn BA, Kraak VI, Allender S, Atkins VJ, Baker PI, Bogard JR (2019). The global syndemic of obesity, undernutrition, and climate change: The Lancet Commission report. The Lancet.

[5] Lobstein T, Jackson-Leach R (2016). Planning for the worst: Estimates of obesity and comorbidities in school-age children in 2025. Pediatr. Obes..

[6] Daniels S (2009). Complications of obesity in children and adolescents. Int. J. Obes..

[7] Han JC, Lawlor DA, Kimm SY (2010). Childhood obesity. The Lancet.

[8] Must A, Anderson SE (2003). Effects of obesity on morbidity in children and adolescents. Nutr. Clin. Care Off. Publ. Tufts Univ..

[9] Daniels SR (2006). The consequences of childhood overweight and obesity. Future Child..

[10] Karelis AD, St-Pierre DH, Conus F, Rabasa-Lhoret R, Poehlman ET (2004). Metabolic and body composition factors in subgroups of obesity: What do we know?. J. Clin. Endocrinol. Metab..

[11] Steinberger J, Daniels SR (2003). Obesity, insulin resistance, diabetes, and cardiovascular risk in children: An American Heart Association scientific statement from the Atherosclerosis, Hypertension, and Obesity in the Young Committee (Council on Cardiovascular Disease in the Young) and the Diabetes Committee (Council on Nutrition, Physical Activity, and Metabolism). Circulation.

[12] Weiss R, Kaufman FR (2008). Metabolic complications of childhood obesity: Identifying and mitigating the risk. Diabetes Care.

[13] Rahimi H, Yuzbashian E, Zareie R, Asghari G, Djazayery A, Movahedi A (2020). Dietary approaches to stop hypertension (DASH) score and obesity phenotypes in children and adolescents. Nutr. J..

[14] Damanhoury S, Newton A, Rashid M, Hartling L, Byrne J, Ball G (2018). Defining metabolically healthy obesity in children: A scoping review. Obes. Rev..

[15] Yngve A, Wolf A, Poortvliet E, Elmadfa I, Brug J, Ehrenblad B (2005). Fruit and vegetable intake in a sample of 11-year-old children in 9 European countries: The Pro Children Cross-sectional Survey. Ann. Nutr. Metab..

[16] Ho M, Garnett SP, Baur LA, Burrows T, Stewart L, Neve M (2013). Impact of dietary and exercise interventions on weight change and metabolic outcomes in obese children and adolescents: A systematic review and meta-analysis of randomized trials. JAMA Pediatr..

[17] Rupérez AI, Mesa MD, Anguita-Ruiz A, González-Gil EM, Vázquez-Cobela R, Moreno LA (2020). Antioxidants and oxidative stress in children: Influence of puberty and metabolically unhealthy status. Antioxidants.

[18] Lauritzen L, Harsløf LB, Hellgren LI, Pedersen MH, Mølgaard C, Michaelsen KF (2012). Fish intake, erythrocyte n-3 fatty acid status and metabolic health in Danish adolescent girls and boys. Br. J. Nutr..

[19] Vuholm S, Rantanen JM, Teisen MN, Stark KD, Mølgaard C, Christensen JH (2019). Effects of oily fish intake on cardiometabolic markers in healthy 8-to 9-y-old children: The FiSK Junior randomized trial. Am. J. Clin. Nutr..

[20] Mirmiran P, Ziadlou M, Karimi S, Hosseini-Esfahani F, Azizi F (2019). The association of dietary patterns and adherence to WHO healthy diet with metabolic syndrome in children and adolescents: Tehran lipid and glucose study. BMC Public Health.

[21] Velázquez-López L, Santiago-Díaz G, Nava-Hernández J, Muñoz-Torres AV, Medina-Bravo P, Torres-Tamayo M (2014). Mediterranean-style diet reduces metabolic syndrome components in obese children and adolescents with obesity. BMC Pediatr..

[22] George ES, Gavrili S, Itsiopoulos C, Manios Y, Moschonis G (2021). Poor adherence to the Mediterranean diet is associated with increased likelihood of metabolic syndrome components in children: The Healthy Growth Study. Public Health Nutr..

[23] Willett W (2012). Nutritional Epidemiology.

[24] Cuenca-García M, Ruiz J, Ortega F, Labayen I, Huybrechts I, Moreno L (2015). A Mediterranean diet is not enough for cardio-metabolic health: Physical activity and physical fitness are major contributors in European adolescent. Rev. Andaluza Med. Deporte.

[25] Yaghoubpour K, Tasdighi E, Abdi H, Barzin M, Mahdavi M, Valizadeh M (2021). Association of obesity phenotypes in adolescents and incidence of early adulthood type 2 diabetes mellitus: Tehran lipid and glucose study. Pediatr. Diabetes.

[26] Onis MD, Onyango AW, Borghi E, Siyam A, Nishida C, Siekmann J (2007). Development of a WHO growth reference for school-aged children and adolescents. Bull. World Health Organ..

[27] Asghari G, Rezazadeh A, Hosseini-Esfahani F, Mehrabi Y, Mirmiran P, Azizi F (2012). Reliability, comparative validity and stability of dietary patterns derived from an FFQ in the Tehran Lipid and Glucose Study. Br. J. Nutr..

[28] Daneshzad E, Ghorabi S, Hasani H, Omidian M, Pritzl TJ, Yavari P (2019). Food Insecurity is positively related to Dietary Inflammatory Index in Iranian high school girls. Int. J. Vitam. Nutr. Res..

[29] Mohseni H, Mohammadi FM, Karampour Z, Amini S, Abiri B, Sayyah M (2021). The relationship between history of dietary nutrients intakes and incidence of aggressive behavior in adolescent girls: A case–control study. Clin. Nutr. ESPEN.

[30] Ghaffarpour M, Houshiar-Rad A, Kianfar H (1999). The manual for household measures, cooking yields factors and edible portion of foods. Tehran Nashre Olume Keshavarzy.

[31] Zimmet P, Alberti G, Kaufman F, Tajima N, Silink M, Arslanian S (2007). The metabolic syndrome in children and adolescents. The Lancet..

[32] Matthews DR, Hosker JP, Rudenski AS, Naylor BA, Treacher DF, Turner RC (1985). Homeostasis model assessment: insulin resistance and beta-cell function from fasting plasma glucose and insulin concentrations in man. Diabetologia.

[33] Prince RL, Kuk JL, Ambler KA, Dhaliwal J, Ball GD (2014). Predictors of metabolically healthy obesity in children. Diabetes Care.

[34] Kurtoglu S, Akin L, Kendirci M, Hatipoglu N, Elmali F, Mazicioglu M (2012). The absence of insulin resistance in metabolic syndrome definition leads to underdiagnosing of metabolic risk in obese patients. Eur. J. Pediatr..

[35] Keskin M, Kurtoglu S, Kendirci M, Atabek ME, Yazici C (2005). Homeostasis model assessment is more reliable than the fasting glucose/insulin ratio and quantitative insulin sensitivity check index for assessing insulin resistance among obese children and adolescents. Pediatrics.

[36] Kowalski KC, Crocker PR, Donen RM (2004). The physical activity questionnaire for older children (PAQ-C) and adolescents (PAQ-A) manual. Coll. Kinesiol. Univ. Sask..

[37] Garmaroudi GR, Moradi A (2010). Socio-economic status in Iran: A study of measurement index. Payesh (Health Monitor).

[38] Kim J-O, Mueller CW (1978). Factor analysis: Statistical methods and practical issues.

[39] Seral-Cortes M, Larruy-García A, Miguel-Etayo PD, Labayen I, Moreno LA (2022). Mediterranean diet and genetic determinants of obesity and metabolic syndrome in european children and adolescents. Genes.

[40] Bozbulut R, Ertaş-Öztürk Y, Döğer E, Bideci A, Köksal E (2020). Increased obesity awareness and adherence to healthy lifestyle-diet reduce metabolic syndrome risk in overweight children. J. Am. Coll. Nutr..

[41] Grosso G, Galvano F (2016). Mediterranean diet adherence in children and adolescents in southern European countries. NFS J..

[42] Zakharova I, Klimov L, Kuryaninova V, Nikitina I, Malyavskaya S, Dolbnya S (2019). Vitamin D insufficiency in overweight and obese children and adolescents. Front. Endocrinol..

[43] Cuadrado-Soto E, López-Sobaler AM, Jiménez-Ortega AI, Aparicio A, Bermejo LM, Hernández-Ruiz Á (2020). Usual dietary intake, nutritional adequacy and food sources of calcium, phosphorus, magnesium and vitamin D of Spanish children aged one to < 10 years. Findings from the EsNuPI Study. Nutrients.

[44] Guo H, Ding J, Liang J, Zhang Y (2021). Associations of whole grain and refined grain consumption with metabolic syndrome. A meta-analysis of observational studies. Front. Nutr..

[45] Asghari G, Yuzbashian E, Mirmiran P, Bahadoran Z, Azizi F (2016). Prediction of metabolic syndrome by a high intake of energy-dense nutrient-poor snacks in Iranian children and adolescents. Pediatr. Res..

[46] Wu Y, Li S, Wang W, Zhang D (2020). Associations of dietary vitamin B1, vitamin B2, niacin, vitamin B6, vitamin B12 and folate equivalent intakes with metabolic syndrome. Int. J. Food Sci. Nutr..

[47] van Dijk SJ, Feskens EJ, Bos MB, Hoelen DW, Heijligenberg R, Bromhaar MG (2009). A saturated fatty acid–rich diet induces an obesity-linked proinflammatory gene expression profile in adipose tissue of subjects at risk of metabolic syndrome. Am. J. Clin. Nutr..

[48] Damsgaard CT, Stark KD, Hjorth MF, Biltoft-Jensen A, Astrup A, Michaelsen KF (2013). n-3 PUFA status in school children is associated with beneficial lipid profile, reduced physical activity and increased blood pressure in boys. Br. J. Nutr..

[49] Gillingham LG, Harris-Janz S, Jones PJ (2011). Dietary monounsaturated fatty acids are protective against metabolic syndrome and cardiovascular disease risk factors. Lipids.

[50] Shahidi F, Ambigaipalan P (2018). Omega-3 polyunsaturated fatty acids and their health benefits. Annu. Rev. Food Sci. Technol..

[51] Simopoulos AP (2008). The importance of the omega-6/omega-3 fatty acid ratio in cardiovascular disease and other chronic diseases. Exp. Biol. Med..

[52] Cascio G, Schiera G, Di Liegro I (2012). Dietary fatty acids in metabolic syndrome, diabetes and cardiovascular diseases. Curr. Diabetes Rev..

[53] Ahima RS, Antwi DA (2008). Brain regulation of appetite and satiety. Endocrinol. Metab. Clin. N. Am..

[54] Artmann A, Petersen G, Hellgren LI, Boberg J, Skonberg C, Nellemann C (2008). Influence of dietary fatty acids on endocannabinoid and N-acylethanolamine levels in rat brain, liver and small intestine. Biochim. Biophys. Acta (BBA) Mol. Cell Biol. Lipids.

[55] Wang Y, Meng X, Deng X, Okekunle AP, Wang P, Zhang Q (2018). Postprandial saturated fatty acids increase the risk of type 2 diabetes: A cohort study in a Chinese population. J. Clin. Endocrinol. Metab..

[56] Micha R, Mozaffarian D (2009). Trans fatty acids: Effects on metabolic syndrome, heart disease and diabetes. Nat. Rev. Endocrinol..

[57] Axen KV, Dikeakos A, Sclafani A (2003). High dietary fat promotes syndrome X in nonobese rats. J. Nutr..

